# Body Mass Index and Exercise Effort Influences Changes in Motor Symptoms After High-Cadence Dynamic Cycling in Parkinson's Disease

**DOI:** 10.3389/fresc.2022.858401

**Published:** 2022-04-15

**Authors:** Peter Gates, Angela L. Ridgel

**Affiliations:** ^1^Motor Control Lab, Exercise Science and Exercise Physiology, School of Health Sciences, Kent State University, Kent, OH, United States; ^2^Brain Health Research Institute, Kent State University, Kent, OH, United States

**Keywords:** movement disorder, rehabilitation, entropy, mixed model analysis, BMI

## Abstract

High-cadence dynamic cycling improves motor symptoms of Parkinson's disease (PD), such as tremor and bradykinesia. However, some participants experience greater benefits than others. To gain insight into how individual characteristics and cycling performance affects functional changes, data from two previous studies were used to build several preliminary predictive models. The purpose was to examine which variables contribute to greater improvement in symptoms after high-cadence dynamic cycling. We hypothesized that individuals with higher body mass index (BMI), increased age, more severe symptoms, and higher PD medication dosages were less likely to contribute effort during cycling. UPDRS-III was assessed before and after each session, and cadence and power were recorded every second. Entropy of cadence was calculated, and data were analyzed using analysis of variance and multiple linear regression. The multiple linear regression model of post UPDRS significantly (R^2^ = 0.81, *p* < 0.001) explained its variance, with pre UPDRS as the main predictor (*p* < 0.0001). The binomial logistic model of mean effort did not significantly (R^2^ = 0.36, *p* = 0.14) explain the variance. *Post-hoc* analysis found a significant (β = 0.28, *p* = 0.03) moderating effect of different levels of BMI on the association between mean effort and post UPDRS. These results suggest that BMI, effort, and baseline UPDRS levels can potentially predict individual responses to high-cadence dynamic cycling.

## Introduction

Parkinson's disease (PD) is the second most common neurodegenerative disease (1) not limited geographically or by age, although prevalence is higher in individuals over 60 (2). In the United States 822/100,000 people over the age of 45 presented with PD in 2010, representing 680,000 individuals. The incidence of PD is projected to rise to more than one million individuals by 2030 (3). Typical symptoms are resting tremors, rigidity, bradykinesia, and postural instability (4). Severity of symptoms increases as degeneration of the substantia nigra progress. Motor symptoms usually appear once 60% of dopaminergic neurons in the substantia nigra have degenerated (5).

Currently available therapeutics do not affect the outcome or progression of the disease but can improve quality of life by reducing motor and non-motor symptoms (6). Pharmaceutical options act to increase the availability of a dopamine precursor, levodopa, or *via* dopamine agonists (6). By increasing dopamine levels in the brain *via* medication, motor symptoms can improve. Prolonged use of these medications can cause adverse side effects such as dyskinesias, nausea, and impulse control (7). Exercise can also help manage symptoms in early and mid-stage PD, potentially allowing for decreased medication dosages and delaying of the need to increase pharmaceutical treatment with progression (8).

Forced cycling, where participants use equipment that “forces” them to pedal at a cadence about 30% above their self-selected level, improves the motor symptoms of PD (9–13) as measured with the Unified Parkinson's Disease Rating Scale part III (UPDRS). The measure is used to assess motor function in PD by analyzing performance on 14 different motor tasks using a scale of 0 (normal) to 4 (severe) (14). An increase in motor function, decreased tremors, and potential promotion of neuroplasticity was previously documented (15). Specifically, a 35% decrease in UPDRS scores was observed with forced cycling but not voluntary solo cycling at the similar heart rate intensity (16). These studies used a stationary tandem bike and an able-bodied trainer to maintain a pedaling cadence of 80-90 revolutions per minute (rpm). A unique feature of the forced cycling session was significant entropy of cadence and power, providing a degree of unpredictability to these measures throughout the duration of the session (13).

The term “high-cadence dynamic cycling” was coined to describe protocols using a motorized cycle that was programed to integrate the degree of unpredictability into the operation of the motor. This dynamic bike was used to examine if motor symptom improvements were similar to that reported after forced cycling. While symptom improvements were statistically and clinically significant in these studies, not all participants experienced them (17, 18).

To understand how individuals respond to high-cadence dynamic cycling, we previously created a measure of effort defined as the percent of power output above zero [(19), Equation 1], that, along with baseline UPDRS, was found to be a significant predictor of UPDRS improvement. We also found that our linear mixed model significantly described the variance in effort of the dataset. However, the independent measures model of effort did not. Because PD is progressive, older individuals usually have more severe symptoms and therefore may show differential responses than younger individuals. In addition, body mass index is associated with increased PD risk (20–22). In light of these findings, we wanted to investigate if other demographic measures, such as age and body mass index (BMI), affected responses to high-cadence dynamic cycling. The current study tested two hypotheses: first that effort, quantified as the percent of time a participant overtakes the dynamic bike motor during the session, is a significant contributor to the variance change in UPDRS, and second that BMI and age are significant contributors to the variance in effort in two additional datasets.

## Methods

Datasets from two previously published repeated measures studies, both showing significant improvements in UPDRS III following high-cadence dynamic bike sessions (17, 18), were used to test our hypotheses. The datasets were merged and correlational, descriptive, and regression statistical methods were completed using the R programming language (23).

### Dataset Description

Dataset 1 (17): 24 participants (12F, 12M, 67 ± 8 years) completed three sessions of high-cadence dynamic cycling across 4 days, with at least 24 h between each session. Cadence was set to 75-85 rpm. Due to the nature of the dynamic bike the cadence varied between that range to maximize sample entropy (SamEn). Each 30-min session was preceded by a 5-min 40-50 rpm warmup and followed up with 5 min of cooldown. During the session participants were encouraged to maintain 50-80% of their estimated heart rate reserves. Pre outcome measures were collected during the first session and post outcome measures were collected twice: first immediately after the last session, and then 48-h after the last session. Pre-post outcome measures included the UPDRS score administered by a blinded movement disorders specialist.

Dataset 2 (18): eight participants (4F, 4M, 70 ± 7 years) completed six 30 min high-cadence dynamic cycling sessions over 2 weeks with 24 h between each session at 75-85 rpm. A 5-min warmup and cooldown at 50 rpm were included in each session for a total of 40-min intervention. Pre-assessment tests (UPDRS) were completed on day 1 before the start of the intervention. These were also done 5 min after each cycling intervention, and once more 48 h after the last intervention. As dataset 1 did not include repeated measures of UPDRS, only the first measure of the first day and the last measure of the last session was included in this study. Participants were encouraged to pedal hard enough for torque values to be positive on the display. Heart rate, cadence, and torque were continuously recorded.

### Protocol

**Figure 1:**
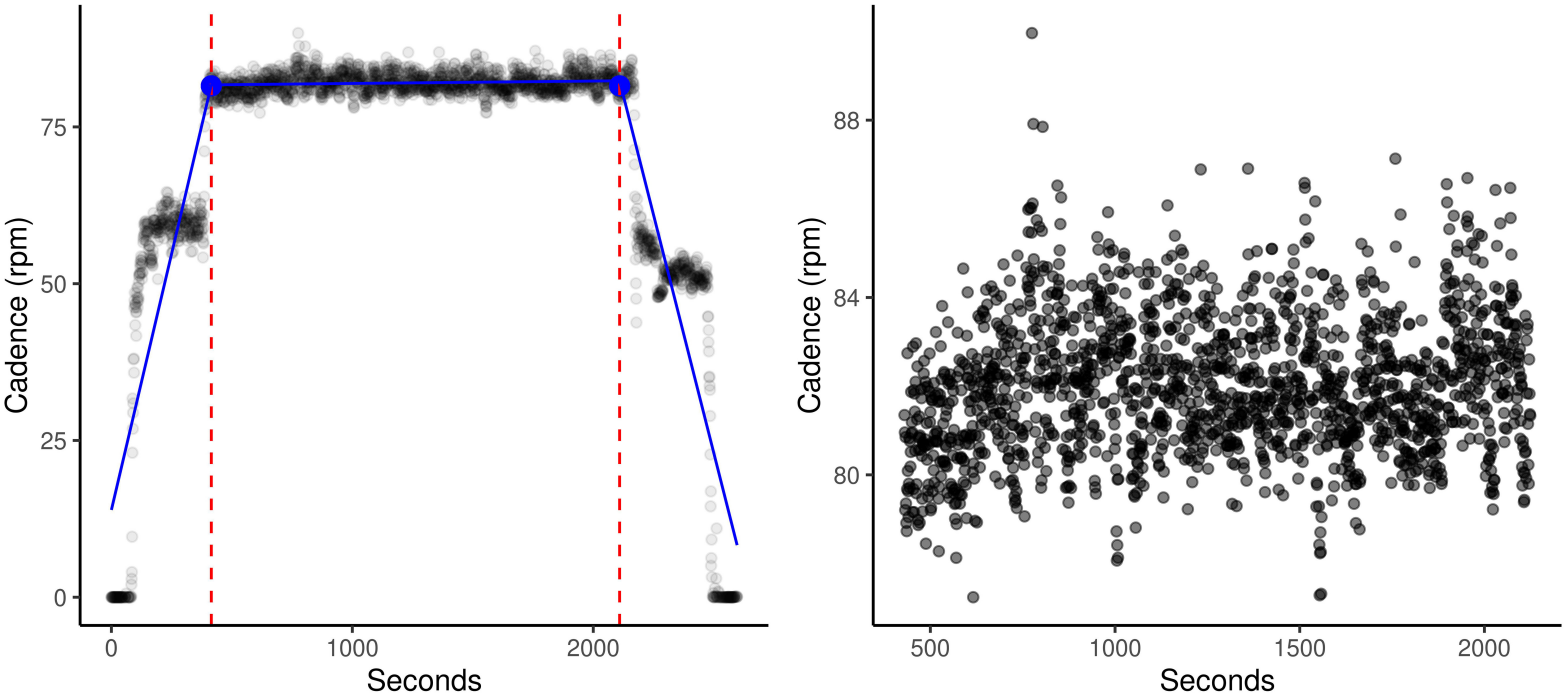
SMB005_day2 (Before) and SMB005_day2 (After). Demonstration of one typical session on the dynamic bike. Dashed lines and blue dot indicate the estimated break points. Blue lines indicate the three calculated regression lines. Using our segment_cutter.r script and the segmented R library, warmups and cooldowns were automatically and objectively cut from the main session. This allowed for more accurate calculations of entropy and effort.

Raw bike output files for each dataset were cleaned and standardized in Python (24), as outlined in the Supplementary Material. As effort is a measure utilizing power output during the main 30-min period of each session, each participant's datasets were run through the segment cutter script to objectively clip warmup and cooldown periods from each session using the segmented library (25–28) in R. This library utilizes segmented regression (piecewise linear regression, stepwise linear regression) to calculate breakpoint (dashed lines, blue dot, Figure 1) and slope estimations of each segmented regression line (blue line, Figure 1). Following extraction of main sessions, the effort for each session was calculated by assigning a value of 1 to each row with a positive power, and 0 for negative power. Each row in the datasets represented one second of collected data. The mean was multiplied by 100 to calculate the percent of time the participant was under positive power, indicating overpowering of the bike motor, during that session.


(1) $$\begin{array}{l} effort={\left(x/N\right)}^{*}100 \end{array}$$

As all outcome variables were measured in the same way between datasets, and any differences can be accounted for with variables representing the dataset or number of sessions, no standardization was needed before the datasets were labeled and merged.

### Outcome Measures

Combined outcome measures utilized from all datasets included pre and post UPDRS scores, cadence in rotations per minute (rpm), heart rate in beats per minutes, and power. The baseline (prior to any cycling session) UPDRS score of each participant is labeled as the pre UPDRS score, and the UPDRS score measured directly after the last biking session was labeled as the post UPDRS score. Demographics collected include height, weight, age, gender, date of diagnosis, medication dosage and frequency. Post study variables were calculated and included the sample (SamEn) and approximate (ApEn) entropies of cadence, effort, body mass index (BMI), and levodopa equivalent dosage (LEDD). The LEDD was calculated with the help of a levodopa equivalent dosage calculator (29), and was included in the model to control for variance in symptom presentation due to medication type and intake. SamEn and ApEn were calculated using a MATLAB script created by Mohammed-Abdar et al. (30), modified to automate the loading of bike output files.

### Data Analysis

Two hypotheses were tested during the study: first that variations in the post UPDRS can be described by effort, preUPDRS scores, daily LEDD, age, number of sessions, and the SamEn of cadence. Number of sessions was included to control for inter-dataset variation and length of exposure to the dynamic bike. Previous studies have shown that UPDRS motor scores continue to improve with greater number of cycling sessions (18). Second, that variations in effort can be described by BMI, preUPDRS scores, daily LEDD, and age. Both were tested with the R version 4.0.5 software to build a multiple linear regression model using the default lm function from the stats (23) library for UPDRS and a binomial logistic model using the glm function from the lme4 (31) library for effort. Linear regression model was chosen as only one of the datasets used included repeated measures. The gvlma library was used to test linear model assumptions (32). The effort model was tested against a null model with the MuMIn (33) library's ANOVA function and the marginal R^2^ was determined using the r.squaredGLMM function from the MuMIn library. Graphs were created using the ggplot2 (34) R package. Alphas were set at <0.05.

## Results

A total of 31 participants' data were used for this analysis, not including one participant from dataset 1 who was removed due to missing demographic data. Mean and standard deviation of key measures are included in Table 1.

**Table 1:** Summary of data used for the model analysis.

	**Dataset 1**	**Dataset 2**	**Combined**
N	24	8	31
Days	3	6	
Age	67 ± 8.1	70 ± 7.4	67.8 ± 7.9
BMI	26.3 ± 4.5	25.7 ± 2.7	26.1 ± 4.1
LEDD	523.3 ± 397.7	422.3 ± 290.7	467.2 ± 371.1
Mean Cadence	78.8 ± 4.1	80.2 ± 2.3	79.1 ± 3.8
SamEn Cadence	1.43 ± 0.4	1.63 ± 0.5	1.5 ± 0.44
Mean Effort	44.5 ± 40.9	51.8 ± 38.2	46.3 ± 39.8
Pre UPDRS	30.4 ± 13.9	14.1 ± 2.1	26.3 ± 13.8
Post UPDRS	26.2 ± 14.2	11.6 ± 1.8	22.6 ± 13.8

*Mean ± standard deviation for each dataset, and then combined. N refers to number of participants used for analysis*.

Dataset 1: During the extraction of main sessions from raw bike sessions, four participants had either day 1 or day 2 sessions removed due to >20 min of collected main session data. One more participant did not have bike data output for day 2. These participants were still included in data analysis, as there was no significant difference in effort, mean cadence, SamEn of cadence, or mean power when tested with RM multivariate analysis of variance (MANOVA) [*F*_(8, 102)_ = 0.96, *p* = 0.97] between sessions for all other participants. One participant took a 2–3-min break during day 2's main session. This session was manually extracted and split, mean power and mean cadence as well as SamEn of cadence calculated separately. The mean of all variables for those two sub-sessions were then entered into the dataset. Effort was calculated as per Equation 1. Paired *t*-test confirmed a significant change in UPDRS scores from day 1 to day 3 (*t* = 3.3, *p* = 0.007).

Dataset 2: One participant's third session was recorded as 0 cadence for the full 45 min, this was regarded as equipment failure and thrown out. The participant's mean effort, power, cadence, SamEn of cadence, were kept for data analysis as there was no significant difference between days for these variables for other participants when tested with RM ANOVA [*F*_(20, 144)_ = 0.60, *p* = 0.91]. Paired *t*-test confirmed a significant difference in UPDRS scores from prescore of day 1 to postscore of day 6 (*t* = 5.2, *p* = 0.002).

Combined Dataset: A significant (r^2^ = −0.44, *p* = 0.02) correlation was found between mean effort, pre UPDRS and post UPDRS, however no other correlations were found (Table 2). Overall, a paired *t*-test found significant difference between the pre UPDRS of the first and post UPDRS of the last day (*t* = 3.63, *p* = 0.0006), and no significant difference was found in post UPDRS scores between the two datasets by a non-parametric ANCOVA test of equality (*h* = 0.76, *p* = 0.06). Due to significant collinearity issues during model building, our outcome variable had to be changed from “change in UPDRS” to post UPDRS. The results from our preliminary unpublished study (19) managed similar collinearity warnings by switching to linear regression with restricted maximum likelihood analysis from maximum likelihood analysis. However that technique did not alleviate the error in this study. To test the hypothesis that the post UPDRS is significantly affected by the pre UPDRS, SamEn of cadence, mean effort, age, daily LEDD, and BMI, a linear model (R^2^ = 0.81, *p* < 0.001) was constructed:


(2) $$\begin{array}{l} \text{updrspost}\text{​}=\text{​ }-8.92\text{​}+\text{​}0.83\text{updrspre}\text{​}-\text{​}0.005\text{meaneffort}\\ \;-5.01\text{samcadence}+0.24\text{age}-2.32\text{med}s_{g}\\ \;+0.11\text{bmi}\text{​}+\text{​}0.03\text{updrspr}e^{*}\text{led}d_{g}\text{​}+\text{​}0.16\text{sessions} \end{array}$$

**Table 2:** Correlation table for the combined dataset.

	**BMI**	**Age**	**LEDD**	**Effort**	**Pre UPDRS**	**Post UPDRS**
BMI	1					
Age	−0.11, *p* = 0.56	1				
LEDD	−0.32, *p* = 0.08	−0.10, *p* = 0.62	1			
Effort	−0.03, *p* = 0.86	−0.30, *p* = 0.10	0.16, *p* = 0.39	1		
Pre UPDRS	−0.03, *p* = 0.89	0.16, *p* = 0.41	0.01, *p* = 0.94	–**0.44**, ***p*** **=** **0.02**	1	
Post UPDRS	0.03, *p* = 0.86	0.23, *p* = 0.23	−0.06, *p* = 0.75	–**0.44**, ***p*** **=** **0.02**	–**0.29**, ***p*** **<** **0.001**	1

*Bolded values are statistically significant. No relationship between gender and effort was found*.

**Figure 2:**
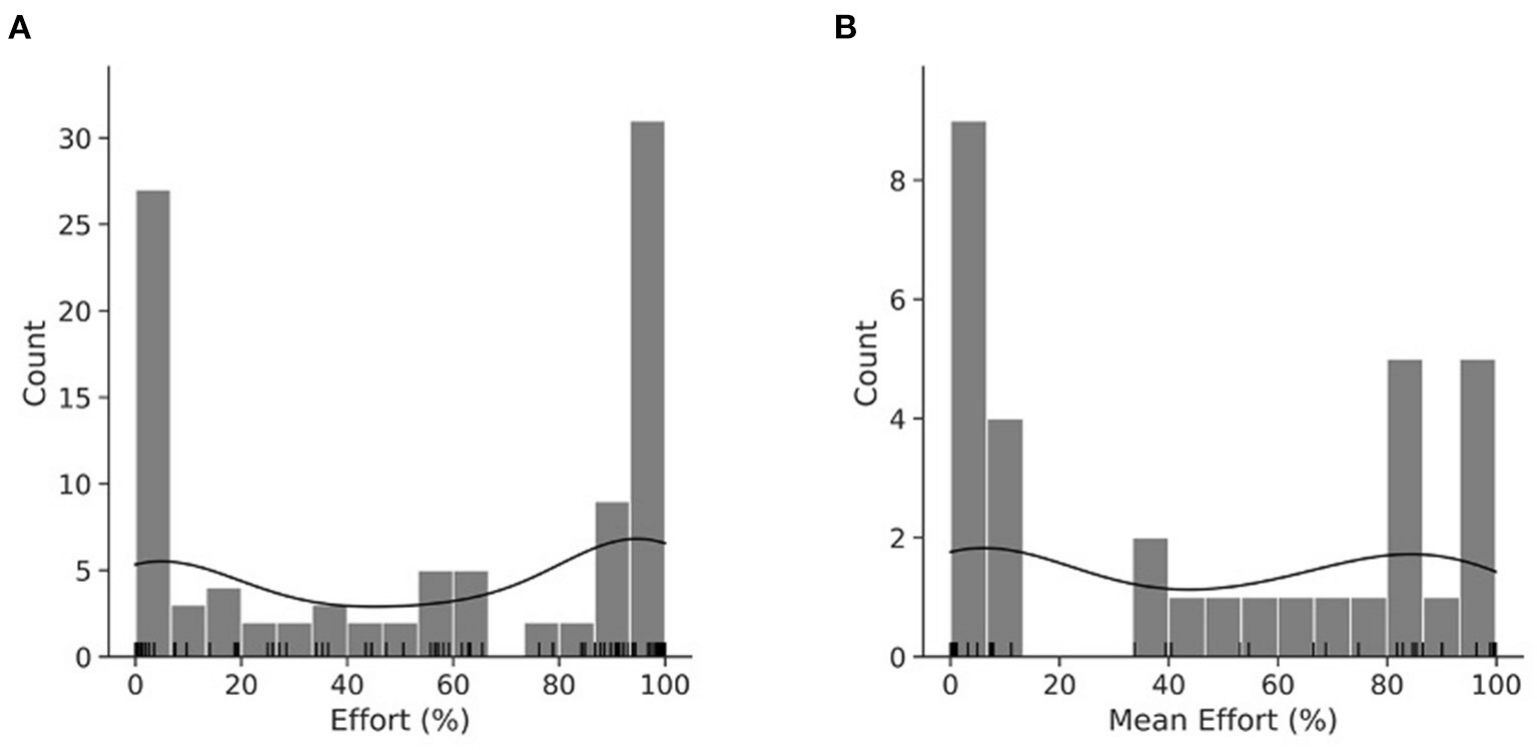
KDE plot of effort per session **(A)**. KDE plot of mean effort per participant **(B)**. Histogram with an overlayed KDE distribution and rug plot demonstrating the binomial nature of effort. **(A)** is the distribution of the RM dataset, while **(B)** is the distribution of mean effort.

Because only the pre UPDRS score was a significant contributor to variance in post UPDRS (*p* < 0.0001), the hypothesis was rejected. Following a review of effort's distribution (Figure 2A shows per session and Figure 2B per participant), the hypothesis that pre UPDRS, age, BMI, and daily LEDD significantly contributed to its variance was slightly modified. While our preliminary study (19) used a linear regression model to describe effort, we designed a binomial general linear model fitted by maximum likelihood with a logit link function. A RM model was not suitable as UPDRS values were not obtained for each day in dataset 1.


(3) $$\begin{array}{l} effort_{bool}=3.2+0.01bmi-0.09age+0.003updrspre\\ \;+3.58ledd_{g}-0.08updrspre^{*}ledd_{g}+0.43sessions \end{array}$$

The mean effort across participant's sessions was categorized into “effort” and “not effort” with 50% as the cutoff point, turning effort into a logical variable. The resulting logistic regression did not show statistically significant predictive power (adj R^2^ = 0.36, *p* = 0.14). To compare our model of effort more easily from the preliminary study (19), a linear model was also tested on the mean effort of participants across sessions.


(4) $$\begin{array}{l} effort_{mean}=\;92.15-\;0.16updrs_{pre}+0.63bmi-1.16age\\ \;+64.43ledd_{g}-1.75updrspre^{*}ledd_{g}+2.64sessions \end{array}$$

Less variance (R^2^ = 0.16, *p* = 0.11) was explained than in our binomial model, there were no significant contributors, and the model was not statistically better from the null hypothesis.

### *Post-hoc* Analysis

We found a minimal contribution by effort (β = −0.005, *p* = 0.94) toward the variance in post UPDRS scores. This minimal contribution was surprising, as our previous study (19) detected a significant contribution to the explained variance (β = −0.1, *p* = 0.04) in updrs_chg, and with the pre UPDRS controlled for in the current model the contribution was expected to have a similar coefficient. Given effort's significant contribution to the repeated measures model but not to the model of means of that study, it may be that an intrapersonal variable that is controlled for by the RM design masked effort's contribution in the current study. The current results show a significant correlation between post UPDRS and mean effort (r^2^ = −0.44, *p* = 0.02). To test whether a moderating variable was involved we ran a *post-hoc* linear regression model with post UPDRS as the dependent variable (DV), mean effort the independent variable (IV), and pre UPDRS as the moderating variable (MoV) (*postUPDRS* = *preUPDRS*+*effort*+preUPDRS^*^effort). There was no significant interaction between pre UPDRS and mean effort and so pre UPDRS was removed from consideration as a MoV.

**Figure 3:**
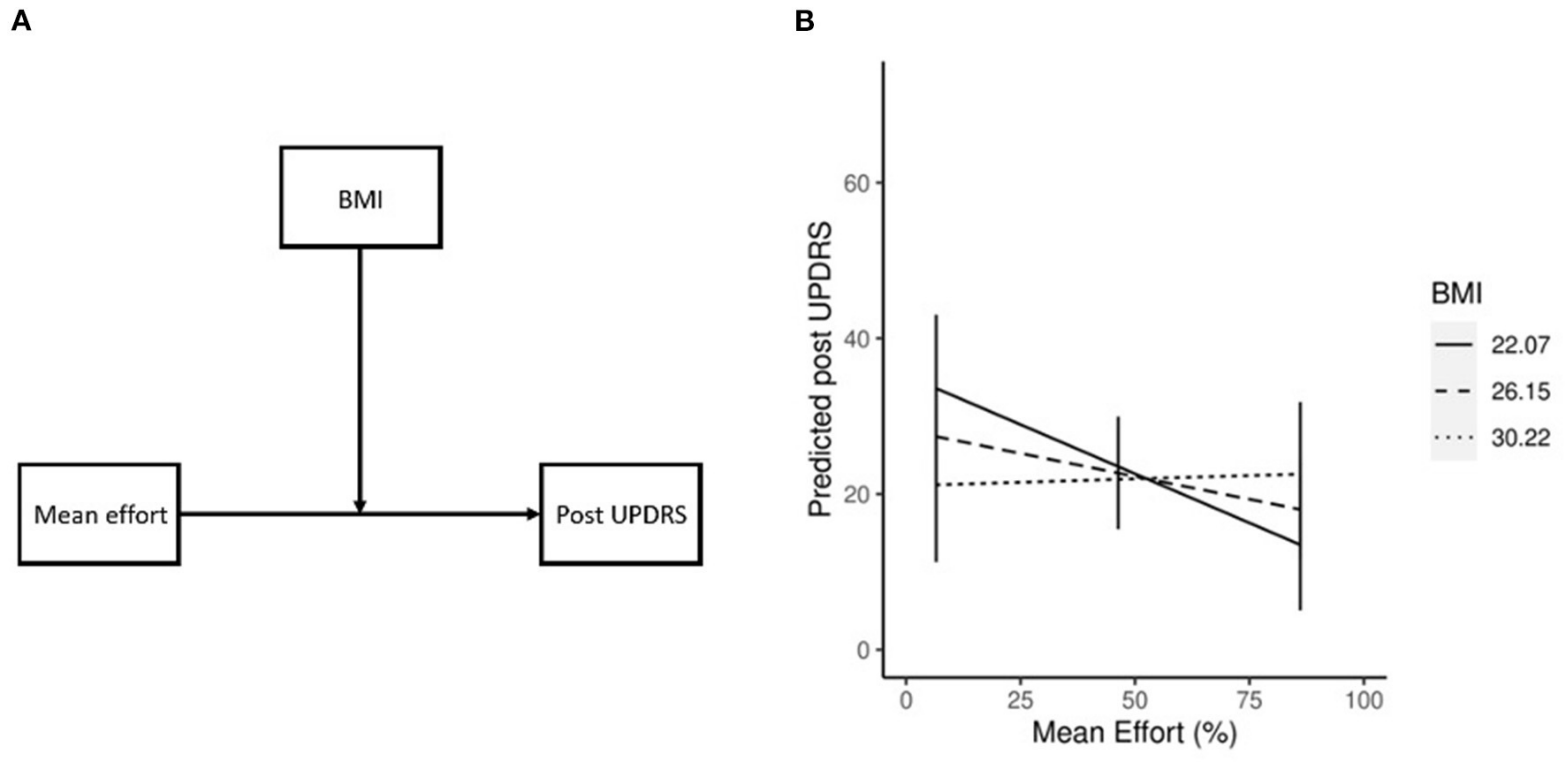
Conceptual diagram of the effect of BMI **(A)**, where the effect of effort on post UPDRS is influenced by BMI. Moderating effect of BMI on mean effort **(B)**. Simple slopes plot demonstrating the moderating effect of BMI on mean effort. As BMI increases the effect of effort on post UPDRS decreases.

Our previous study (19) showed a significant correlation between BMI and effort (r^2^ = −0.72, *p* = 0.01, chapter 2), and while no significant correlation was shown in the current study it is possible that BMI had a moderating effect on mean effort. To test this, we *post-hoc* ran a linear regression model with post UPDRS as the DV, mean effort as IV, and BMI as MoV (*postUPDRS* = *bmi*+*effort*+bmi^*^effort). We found a significant contribution of the interaction between IV and MoV (β = 0.28, *p* = 0.03) to the post UPDRS, indicating a moderating effect of different levels of BMI on the association of mean effort and post UPDRS (Figure 3A). The BMI (β = −1.74, *p* = 0.08) and mean effort (β = −0.98, *p* = 0.02) both contributed to the post UPDRS. To better understand the relationship between these variables the estimated marginal means and simple slopes were calculated using the emmeans (35) R library. Our results (Figure 3B) show that with increasing levels of BMI, one unit increase in the mean effort is associated with improvements in the post UPDRS score where a lower score indicates less severe motor symptoms. At low BMI (22.07, or one std below the mean of our dataset) the post UPDRS decreased by 0.25 for each unit increase in effort. The mean BMI (26.15) showed similar results, where each unit increase in effort was associated with a 0.11 decrease in post UPDRS. High BMI (30.22, or one std above the mean) did not see much effect at a 0.02 increase in post UPDRS.

**Figure 4:**
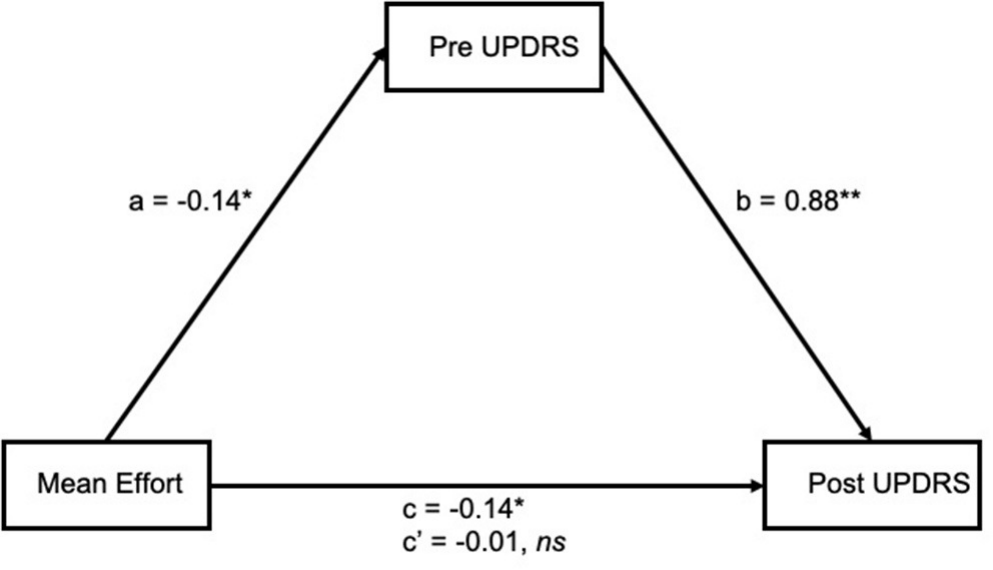
Mean effort had a significant association with both pre (a) and post (c) UPDRS, and pre UPDRS with post UPDRS (b). The effect between mean effort and post UPDRS was fully mediated by pre UPDRS (c'). *p < 0.05 **p < 0.01.

While no moderating relationship was found between pre UPDRS and mean effort, a mediating relationship is also likely. If pre UPDRS is a mediator of mean effort, the possibility that pre UPDRS is a mediator of other variables can be explored, such as the participant's power output (a measure similar to, but more direct than, effort) or cadence (the dynamic bike calculates SamEn from a preset range of cadence, however not all participants are able to keep up with that cadence). The four step method of mediation analysis as outlined by Baron and Kenny (36) was used, along with the R package mediation (37) to test for significance. Our results (Figure 4) showed a significant association between mean effort and post UPDRS (c = −0.14, *p* = 0.02) and pre UPDRS (a = −0.14, *p* = 0.02). There were also significant associations between pre UPDRS and post UPDRS (b = 0.88, *p* < 0.001). As both a and b relationships were significant, pre UPDRS was considered a mediator. The effect of mean effort on post UPDRS was fully mediated by pre UPDRS (c' = −0.01, *p* = 0.69). The unstandardized indirect effects were found for each of 1,000 bootstrapped samples and averaged together, then confidence intervals determined at the 2.5th and 97.5th percentiles. This mean indirect effect was −0.14 (−0.24, −0.03) and determined to be statistically significant (*p* = 0.04).

## Discussion

The primary goal of this analysis was to identify participant characteristics or demographic variables that might influence the degree of UPDRS change following several sessions of high-cadence dynamic cycling. We demonstrated an automated and objective way of extracting main sessions from between warmup and cooldown times using our segment cutter script (Supplementary Data). We also confirmed a significant change in UPDRS scores between the first and last session of each dataset, and no significant changes in effort between sessions. This latter finding lends further support to the idea that effort, defined as the percent of time a participant overtakes the bike motor, is a result of properties intrinsic to each participant and not necessarily to the bike. We also tested two models, one of UPDRS (Equation 2) and another of effort (Equation 3), to describe the changes seen in both datasets. The former explains variance in its outcome measure significantly better than the null hypothesis, however the latter does not. The UPDRS model explains 81% of variance in our participants' post UPDRS scores, with the main significant contributor being baseline UPDRS scores. The finding suggests that participants with more severe symptoms are more likely to improve. Effort's contribution was minimal, with a coefficient of −0.005 (the variable effort was in decimal, not percent form), and was not significant.

The relationship between baseline UPDRS, post UPDRS, and effort indicate the importance of baseline UPDRS or symptom severity in participant's response to high-cadence dynamic cycling. There was also a moderating relationship between BMI and effort. This finding lends support to our previous observation that participants in the obese category of BMI provided low effort (<35%) regardless of UPDRS score, while those in the normal category of BMI were capable of high effort (>65%) with lower UPDRS score (19). Similarly, following our correlation results the moderation analysis found a decreased effect of effort on post UPDRS based with increasing BMI. A concrete statement cannot be made based off these observations alone. It is likely that our independent measures design is not accounting for individual variance, thereby masking our results. Further testing is needed to decipher the precise relationship between demographic variables, effort, and UPDRS outcome. By identifying these relationships, it is possible to adapt the high-cadence dynamic cycling prescription to individual characteristics and maximize the outcome.

Our models of effort suggest a True/False property, where effort may be best described not on a continuous but a binomial scale. The current definitions of >50% of time in positive power is “effort” and <50% is “no effort” is slightly different from our previous reporting (19) that >65% is “high” and <35% is “low” effort. It is likely that the low sample size of 16 participants in the previous study did not sufficiently capture the pattern as our current n=31. Unlike the previous model, our current model of mean effort (Equation 4) and the log probability of expending effort (Equation 3) did not explain variance in the outcome to a statistically significant degree. This might be due to the repeated measures design of our previous model that can account for interpersonal variance unlike our current independent measures-based model. The fact that the previous linear model of mean effort also did not show significance supports this idea. More studies are needed to conclusively determine the role of effort on the post UPDRS score and the role of demographic variables on effort. Previous studies have shown that intensity of exercise, as defined by heart rate, can drive motor function improvements, and potentially promote neuroplasticity (38). However, heart rate is not necessarily a direct measure of effort in this population due to autonomic dysfunction (39) and chronotrophic incompetence (40).

One limitation of this study is lack of heart rate as a variable in the model due to missing or inaccurate data. However, previous cycling studies have shown that heart rate is not a direct predictor of improvement in UPDRS III scores after high-cadence cycling (16, 17), so it is not likely to contribute to the model. The next step is to collect additional data over a longer exercise period (12 sessions) with a larger sample. The significant improvements in UPDRS scores after high-cadence dynamic cycling is promising but the variance between individuals was considerable. Future studies will utilize our script to extract main sessions and develop more accurate and valid models of motor symptoms changes after high-cadence dynamic cycling. By understanding what variables influence this interaction, the cycling-based exercise prescriptions can be tuned to better accommodate participants with certain intrinsic or performance-based characteristics. As our dataset of results and variables grow, a machine learning model can be implemented that may improve the prediction power of our models and better describe the parameters that can most influence improvements in UPDRS scores after high-cadence cycling.

## Conclusion

These results suggest that BMI, physical effort, and baseline UPDRS levels can potentially predict individual responses to high-cadence dynamic cycling. Future studies improve upon this model to develop an adaptive controller that will optimize symptom improvements in individuals with PD.

## Data Availability Statement

The raw data supporting the conclusions of this article will be made available by the authors, without undue reservation.

## Ethics Statement

The studies involving human participants were reviewed and approved by Kent State University Institutional Review Board. The patients/participants provided their written informed consent to participate in this study.

## Author Contributions

PG developed the equations, completed the analysis, and drafted the manuscript. AR supervised the study. Both authors contributed to conception and design of the work and approved the submitted version.

## Funding

This work was supported by the Davis Phinney Foundation, TeCK Fund, and a Mid-Career EHHS SEED Award to AR.

## Conflict of Interest

AR is a co-inventor on two patents which are related to the device used in this study: “Bike System for Use in Rehabilitation of a Patient,” US 10,058,736 and US 9,802,081. No royalties have been distributed from this patent. The remaining author declares that the research was conducted in the absence of any commercial or financial relationships that could be construed as a potential conflict of interest.

## Publisher's Note

All claims expressed in this article are solely those of the authors and do not necessarily represent those of their affiliated organizations, or those of the publisher, the editors and the reviewers. Any product that may be evaluated in this article, or claim that may be made by its manufacturer, is not guaranteed or endorsed by the publisher.
