# Supplementary material for: Body Mass Index and Exercise Effort Influences Changes in Motor Symptoms After High-Cadence Dynamic Cycling in Parkinson's Disease
Source: Front Rehabil Sci. 2022 Apr 15;3:858401. doi: 10.3389/fresc.2022.858401 (PMC9397762; doi:10.3389/fresc.2022.858401)
Supplement: Supplementary file 1 [file Data_Sheet_1.pdf]

Supplementary Material

1 Supplementary Data

1.1 Processing and organizing raw and formatted datasets

Datasets 1 and 2 were organized using the python script raw_processing.py, the full code of which can be accessed on the GitHub page at https://github.com/pomkos/dynamic_cycling_paper_2022. The dfBike class was used to clean and organize raw bike output files. The dfDemos class was used to calculate effort from each participant's dfBike file, **Equation 1** was utilized. The resulting dataset was merged with demographic and overall UPDRS data to create the dfDemos dataset, including means of relevant repeated measures as needed. Finally, the dfEntropy class was used to organize previously calculated entropies in a standardized manner.

1.2 Automated clipping of warmup and cooldown periods with piecewise regression

The following script segment_cutter.r created in R automates the clipping of warmup and cooldown periods of datasets previously formatted with the raw_processing.py script. This was done with the help of the segmented package. This package uses piecewise regression to calculate where the breakpoint for each regression line exists on the x-axis. The only required input for the function is the dataset itself and the estimated number of cuts each dataset requires. The full code can be accessed at

1.3 Example extraction of session with intermittent breaks

Optimal points were selected in python using the Plotly[35] library. The criteria used:

1. Selected observation must be the closest point to the "main session"
2. Data point immediately before/after the selected observation, depending on the cut, must jump far outside the "main session"

The resulting cut is shown in **Supplementary Figure 1A**, with selected observation values in red. The two sub datasets were analyzed separately as entropy analysis assumes a continuous time series. As the time series was broken, the sub datasets were analyzed as if they were separate (**Supplementary Figure 1B**). The resulting entropies were then averaged before further analysis was done. The same procedure was used for descriptive statistics of cadence, power, heart rate.

2 Supplementary Figures and Tables

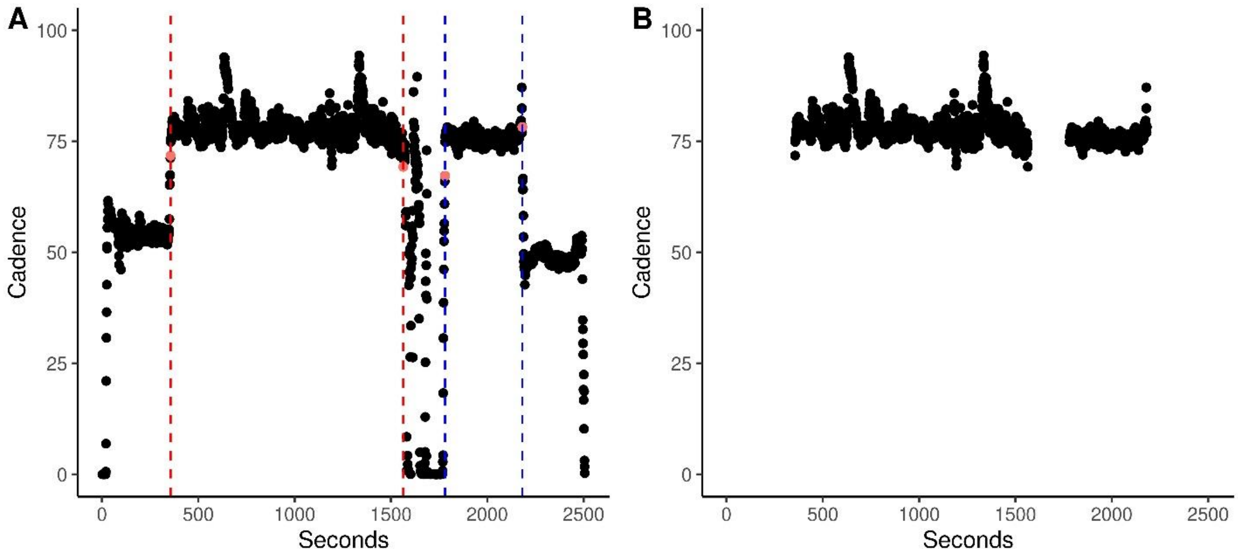

Supplementary Figure 1. *pddy002, Day 4 (Before)* (A) Participant took a break at approximately 1500 seconds. The dataset was manually cut along the dashed lines and separated into part 1 (red) and part 2(blue). Red datapoints represent selected cutoff values. *pddy002, Day 4 (After)* (B) Final results of the manual cut.
